# Supplementary material for: The Neuroprotective Potential of Seed Extract from the Indian Trumpet Tree Against Amyloid Beta-Induced Toxicity in SH-SY5Y Cells
Source: Int J Mol Sci. 2025 Jun 29;26(13):6288. doi: 10.3390/ijms26136288 (PMC12250397; doi:10.3390/ijms26136288)
Supplement: Supplementary file 1 [file ijms-26-06288-s001.zip › ijms-3709159-supplementary.pdf]

Originate for figure 2A (control)

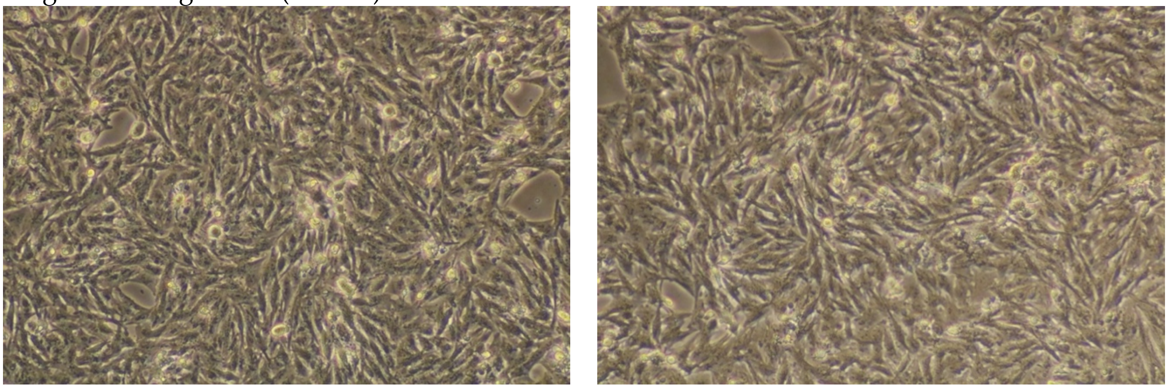

Originate for figure 2A (Aβ)

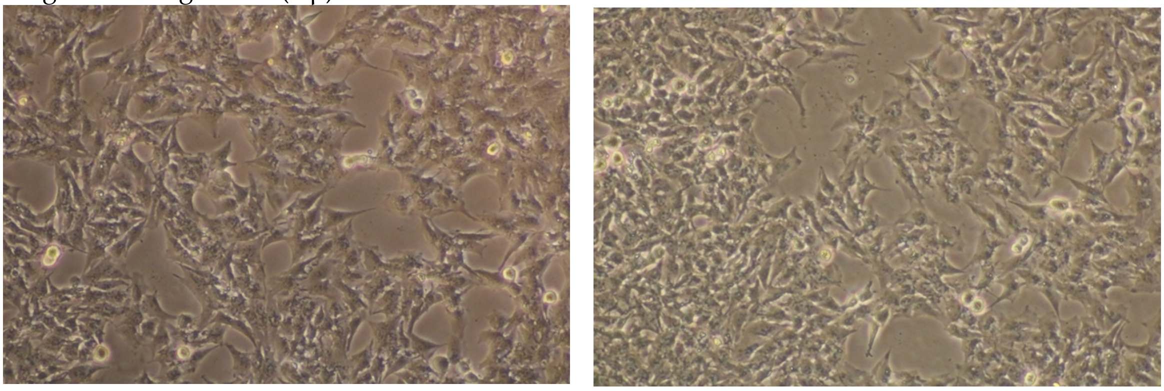

Originate for figure 2A (Aβ + ITS 25 μg/mL)

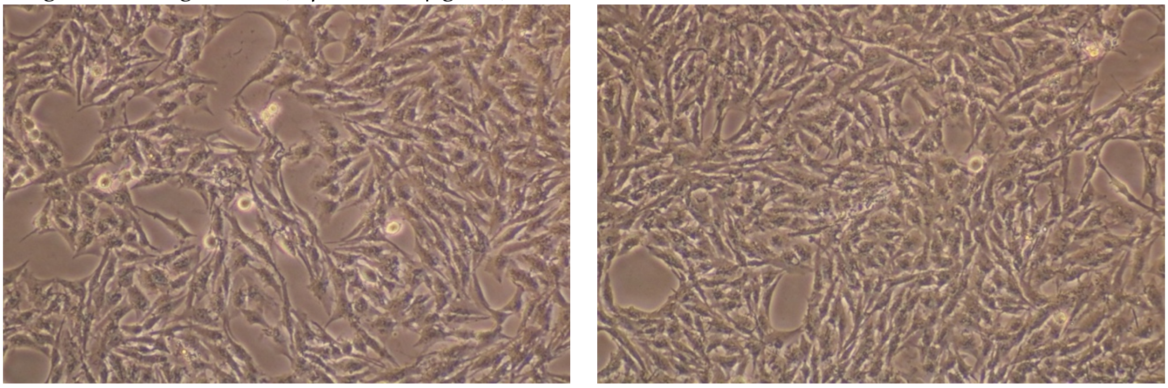

Originate for figure 2A (Aβ + ITS 25 μg/mL)

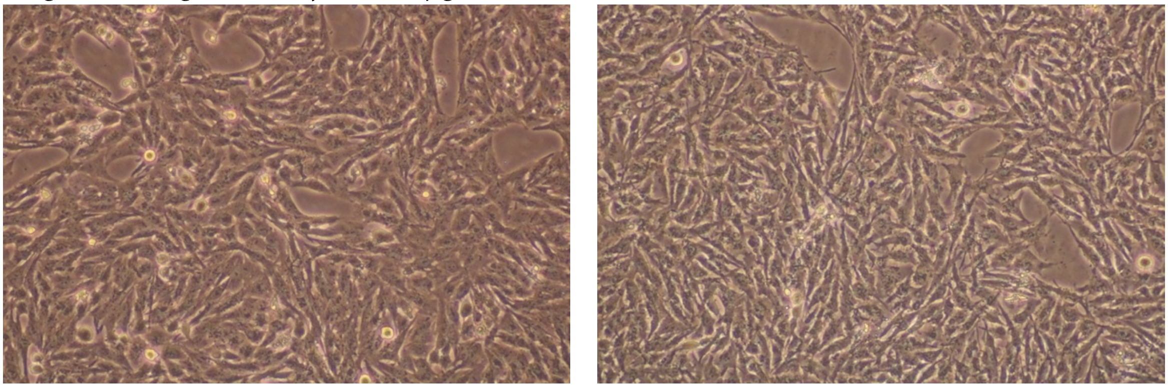

Figure S1: Light microscopy images showing SH-SY5Y cell morphology at 10× magnification. Experimental groups included: (1) control, (2) Aβ, (3) Aβ + ITS (25 μg/mL), and (4) Aβ + ITS (25 μg/mL). Abbreviations: Aβ, amyloid beta; ITS, seed extract of the Indian trumpet tree.

Originate for figure 4

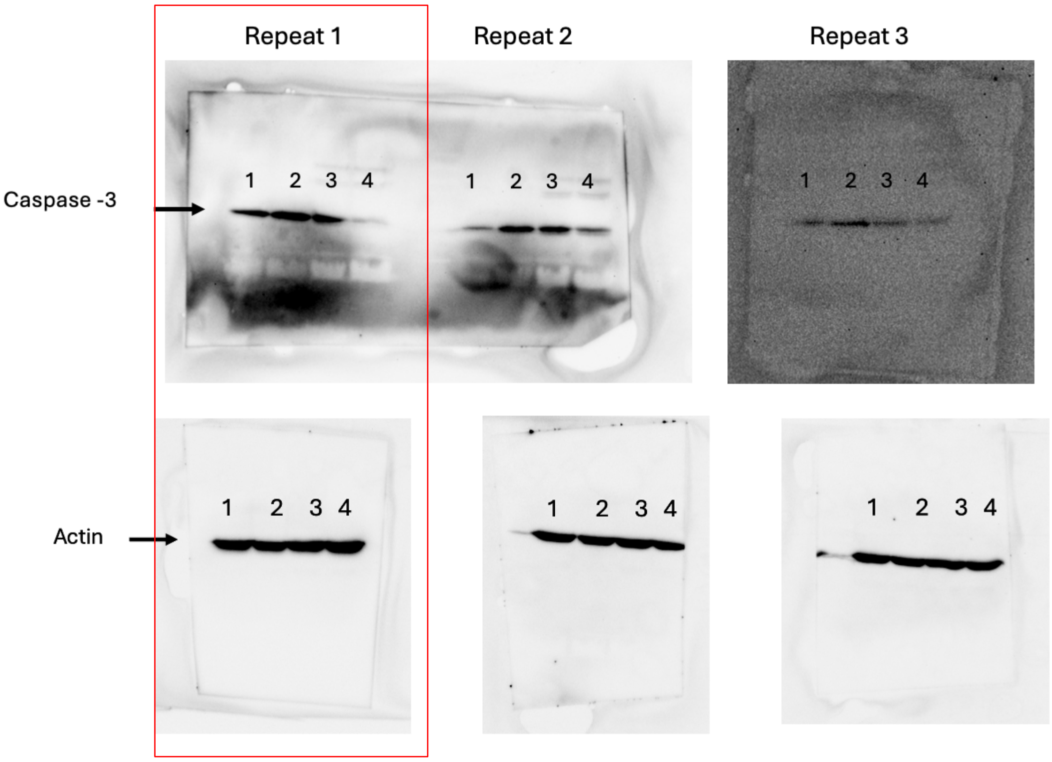

Figure S2: Full-length, uncropped Western blot membranes showing caspase-3 expressions in SH-SY5Y cells under Aβ-induced cytotoxicity. Experiments were repeated three times, and the image highlighted with a red box represents the blot shown in the manuscript. The experimental conditions were as follows: (1) control, (2) Aβ, (3) Aβ + ITS (25 μg/mL), and (4) Aβ + ITS (25 μg/mL). Abbreviations: Aβ, amyloid beta; ITS, seed extract of the Indian trumpet tree.

Originate for figure 5A

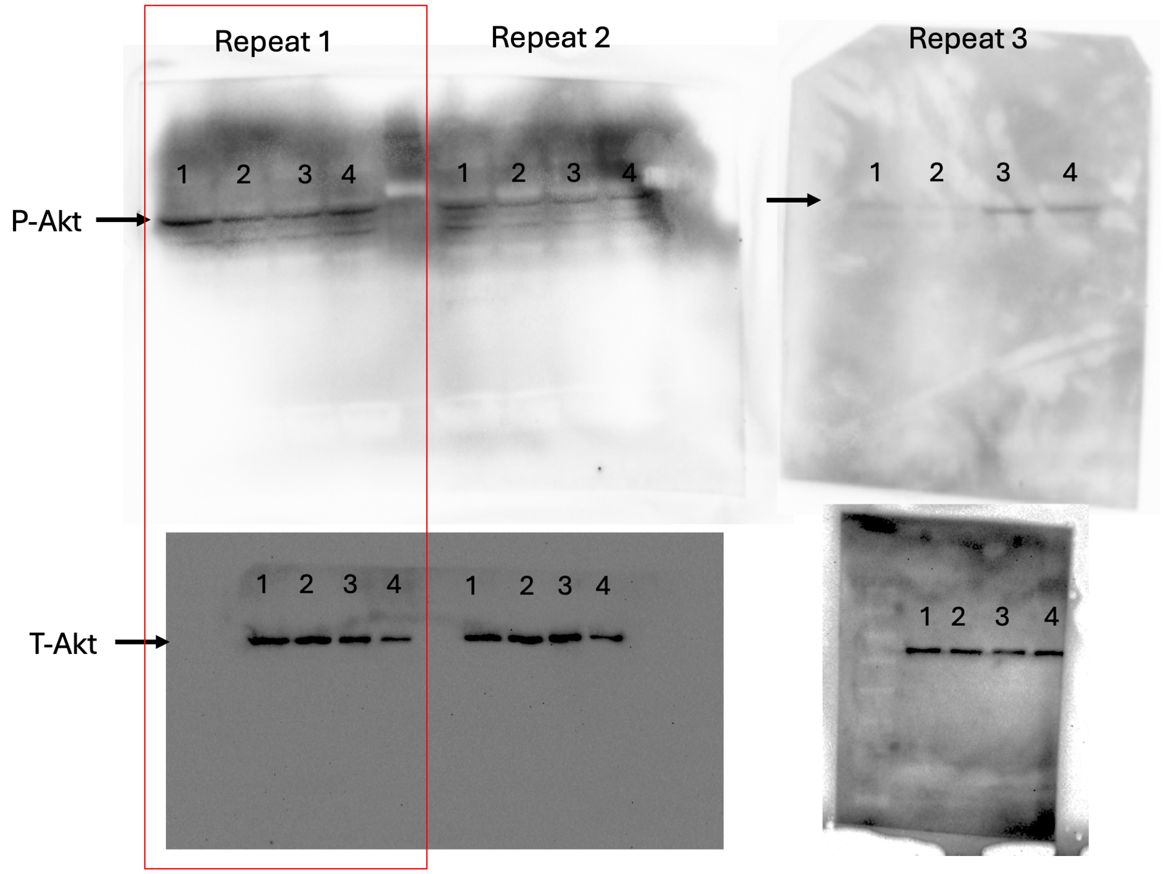

Figure S3: Full-length, uncropped Western blot membranes showing p-Akt and T-Akt expressions in SH-SY5Y cells under Aβ-induced cytotoxicity. Experiments were repeated three times, and the image highlighted with a red box represents the blot shown in the manuscript. The experimental conditions were as follows: (1) control, (2) Aβ, (3) Aβ + ITS (25 μg/mL), and (4) Aβ + ITS (25 μg/mL). Abbreviations: Aβ, amyloid beta; ITS, seed extract of the Indian trumpet tree.

Originate for figure 5B

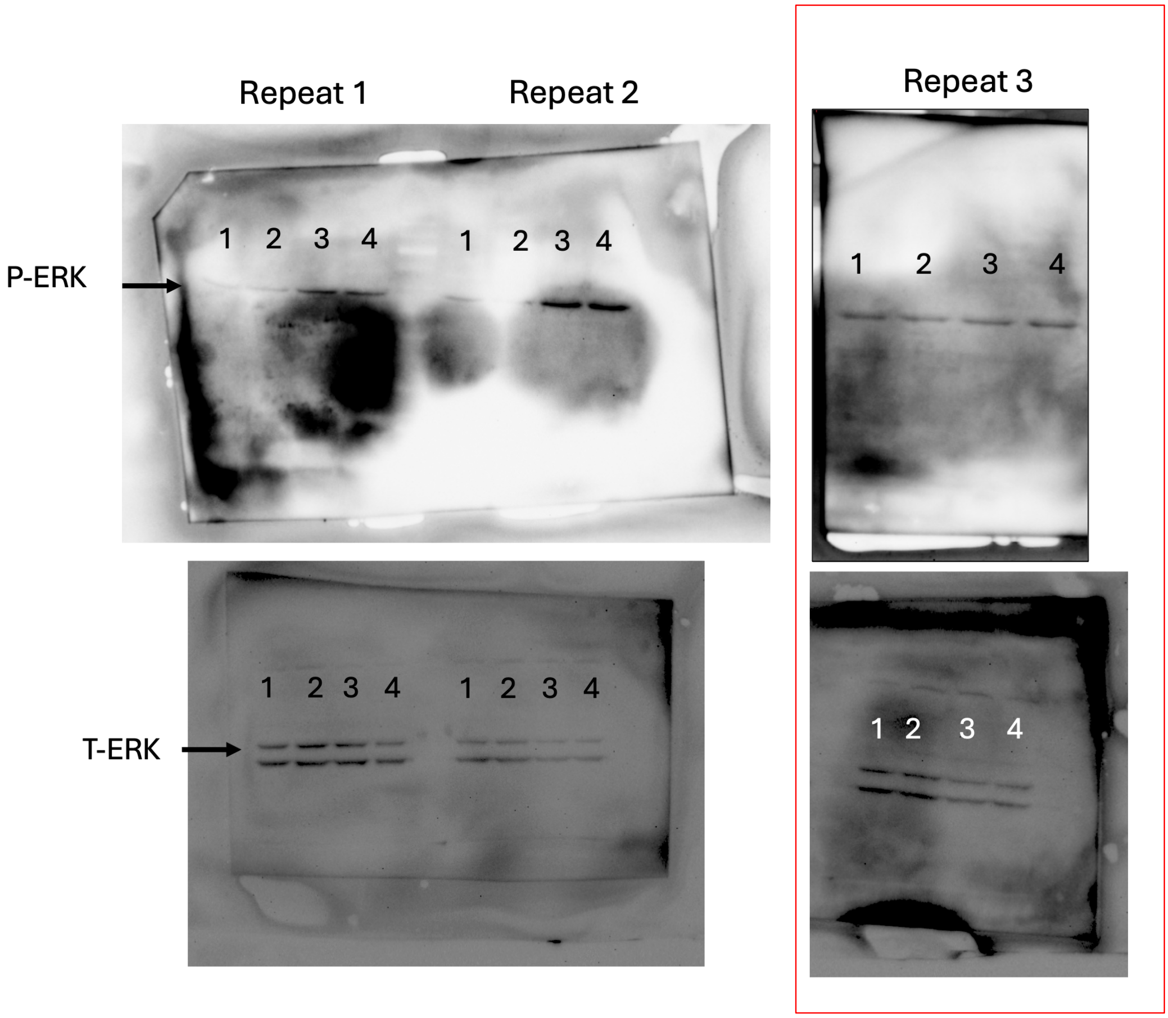

Figure S4: Full-length, uncropped Western blot membranes showing p-ERK and T-ERK expressions in SH-SY5Y cells under Aβ-induced cytotoxicity. Experiments were repeated three times, and the image highlighted with a red box represents the blot shown in the manuscript. The experimental conditions were as follows: (1) control, (2) Aβ, (3) Aβ + ITS (25 μg/mL), and (4) Aβ + ITS (25 μg/mL). Abbreviations: Aβ, amyloid beta; ITS, seed extract of the Indian trumpet tree.

Originate for figure 6A

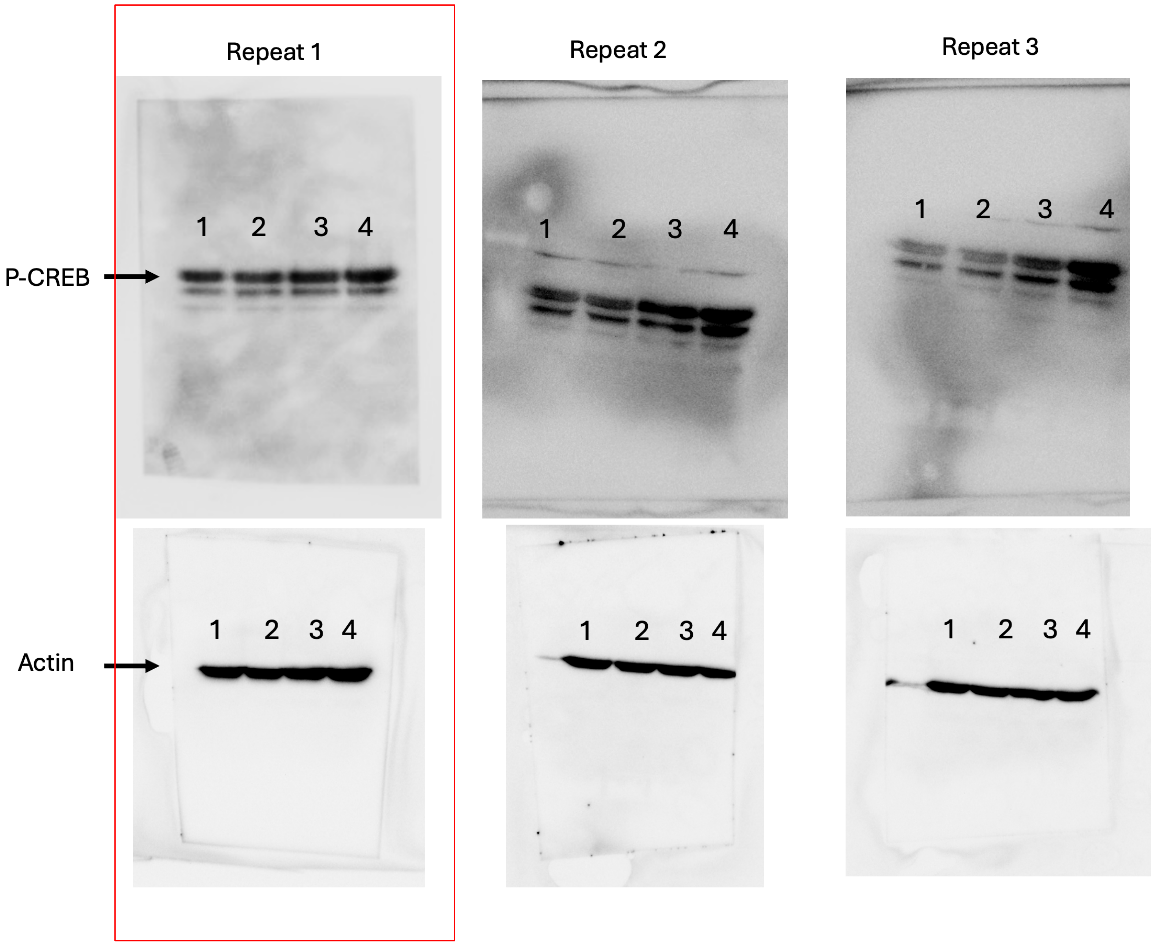

Figure S5: Full-length, uncropped Western blot membranes showing p-CREB expressions in SH-SY5Y cells under Aβ-induced cytotoxicity. Experiments were repeated three times, and the image highlighted with a red box represents the blot shown in the manuscript. The experimental conditions were as follows: (1) control, (2) Aβ, (3) Aβ + ITS (25 μg/mL), and (4) Aβ + ITS (25 μg/mL). Abbreviations: Aβ, amyloid beta; ITS, seed extract of the Indian trumpet tree.

Originate for figure 6B

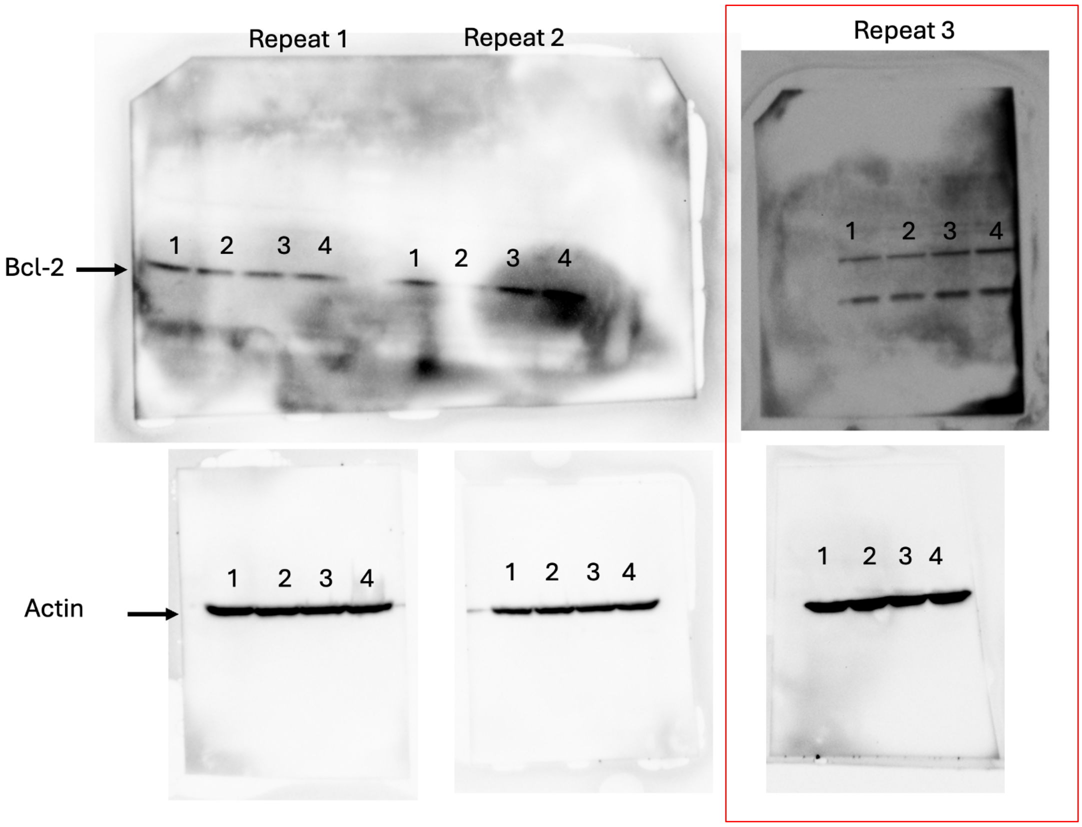

Figure S6: Full-length, uncropped Western blot membranes showing Bcl-2 expressions in SH-SY5Y cells under Aβ-induced cytotoxicity. Experiments were repeated three times, and the image highlighted with a red box represents the blot shown in the manuscript. The experimental conditions were as follows: (1) control, (2) Aβ, (3) Aβ + ITS (25 μg/mL), and (4) Aβ + ITS (25 μg/mL). Abbreviations: Aβ, amyloid beta; ITS, seed extract of the Indian trumpet tree.
